# Visual and thermal camouflage on different terrestrial environments based on electrochromism

**DOI:** 10.1515/nanoph-2023-0244

**Published:** 2023-06-12

**Authors:** Suwan Jeon, Su Eon Lee, Wonjoong Kim, Sun Hee Lee, Seokhwan Min, Seung Won Seon, Seung Ho Han, Bong Hoon Kim, Heon Lee, Jonghwa Shin

**Affiliations:** Department of Materials Science and Engineering (DMSE), Korea Advanced Institute of Science and Technology (KAIST), Daejeon 34141, Republic of Korea; Department of Nano Mechanics, Korea Institute of Machinery and Materials (KIMM), Gajeongbuk-ro 156, Yuseong-gu, Daejeon 34103, Republic of Korea; Department of Robotics and Mechatronics Engineering, Daegu Gyeongbuk Institute of Science and Technology (DGIST), Daegu 42988, Republic of Korea; Department of Materials Science and Engineering (DMSE), Korea University, Anam-ro 145, Seongbuk-gu, Seoul, 02841, Republic of Korea; Electronic Convergence Materials & Device Research Center, Korea Electronics Technology Institute (KETI), Seongnam 13509, Republic of Korea; Department of Smart Wearable Engineering, Soongsil University, Seoul 06978, Republic of Korea

**Keywords:** camouflage, dual bands, dual environments, electrochromism, radiative cooling

## Abstract

Hiding terrestrial objects from aerial monitoring has long been an important objective in national security and public safety. However, the diversity of terrestrial environments raises great challenges to traditional camouflages optimized for a single spectral band or single type of background environment, rendering them vulnerable in other bands or backgrounds. Herein, we experimentally demonstrate simultaneous visual and thermal camouflage that can adapt to two different environments based on a thermally emissive electrochromic layer. We first explore diverse possible theoretical solutions for dual-band dual-environmental camouflage by solving analytic constraints for camouflage and steady-state thermal conditions and select the most viable approach. Based on the theoretical analysis, we design active camouflage thin-film material systems that can approximate two different target visible and infrared signatures of backgrounds under proper bias voltage. Our potentially flexible camouflage surfaces can also conceal heat sources such as human body as well with tailored designs. These results provide new directions in multi-band stealth designs.

## 1 Introduction

The demand for camouflage, an essential survival tactic of prey against predators in nature, is rapidly increasing as a means of protecting important public assets and privacy of individual against fast-evolving surveillance and reconnaissance systems, such as unmanned aerial vehicles (UAVs) and commercial satellites [1]. However, traditional camouflages are typically designed for a particular spectral band and susceptible to modern detection technologies employing multispectral sensing [2, 3], high spatial-temporal resolution [4, 5], and AI-assisted inspection [6, 7]. In principle, multi-band sensing can be easily achieved just by combining different sensors in different bands (though not ideal in terms of system form factor and operability). By contrast, camouflaging across all relevant bands cannot be accomplished, for example, just by layering different camouflage surfaces together because each surface has emission and reflection signatures at all bands and can significantly deteriorate or even nullify the camouflage performance outside the single band the particular layer is optimized for. Thus, developing multi-band camouflage surfaces is a difficult task that requires synthesis of new materials or metamaterials with precisely designed electromagnetic properties across all relevant bands, which span from hundreds of nanometers to a few millimeters in wavelengths (e.g., color in the visible region, radiation intensity in infrared (IR) region, and radar cross-section in radio frequency) [8].

The difficulty escalates even further if the camouflage system has to adapt to more than one environment in real-time like chameleons [9] as it would require tunability of such sophisticated electromagnetic properties. Unlike aerospace, terrestrial environments exhibit large variance in the visible and infrared characteristics due to numerous types of materials involved, such as soil, rock, snow, and vegetation [10, 11]. Thus, for the effective concealment of terrestrial objects, their exterior must adapt to diverse radiative signatures of the spatially and temporally varying environments. However, conventional camouflages have fixed properties optimized to an average setting, which limits their efficacy in such changing environments.

To resolve the above issues, recent studies have extensively attempted to expand the camouflage performance into multiple bands [8, 12], [13], [14], [15] or multiple backgrounds [16, 17] with a single device. For multi-band camouflage, the heterogeneity of the required properties over wide bands were partially reconciled by precisely designing photonics crystals [18, 19], metasurfaces [20, 21], and nested structures with different spectral objects [8, 22, 23] to imitate radiative signatures of the target environment. In addition, the switchable radiative function has been studied for the concealment in varying environments or on-off control of the concealment, based on electrochromism [24–26], phase-change materials [27, 28], and physical transformation [17, 29, 30]. Nevertheless, the all-around camouflage for wide bands and diverse backgrounds has not been realized yet.

In this study, we design and demonstrate an electrically controllable dual-band camouflage thin-film system that can adapt visually and thermally to different ground-like environments. With theoretical analysis of the principle of camouflage, we explicate that the concealments in visible and IR ranges are intertwined processes where the thermal load from the sun has fundamental upper and lower bounds determined by the object’s desired visual appearance (by metamerism), which, in turn, sets the bounds for its IR emittance in order to match the thermal signature with surroundings. An important result of the analysis is the existence of multiple steady-state solutions for dual-band camouflage. Based on this, we design a physically realizable dual-band camouflage system that can adapt to two different backgrounds with very different colors but similar IR emittances like typical terrestrial environments. The key idea for design is to sustain large IR emittance while changing the perceived color. Following this design approach, we build an active dual-band camouflage device by stacking an electrochromic layer with a visibly transparent layer that exhibits consistently high emittance in 8–13 μm (*ε*_8–13_ ≳ 83 % and Δ*ε*_8–13_ ≲ 1 %) wavelength range. The system can show a significant change in its exterior color (Δ*E*_00_ ≳ 47 at D65 standard illuminant) with bias voltages. The outdoor experiments verify that the active camouflage device is visually and thermally concealed against two backgrounds and that the principle remains valid even when concealed objects internally generate moderate level of heat (∼50 Wm^−2^). We also note that the device can be made flexible by choosing polymeric layers as emissive layers, potentially applicable to chameleon-like wearable camouflages or rollable canopies.

## 2 Results and discussion

### 2.1 Visual and thermal conditions for steady-state camouflage in a fixed environment

**Figure 1:**
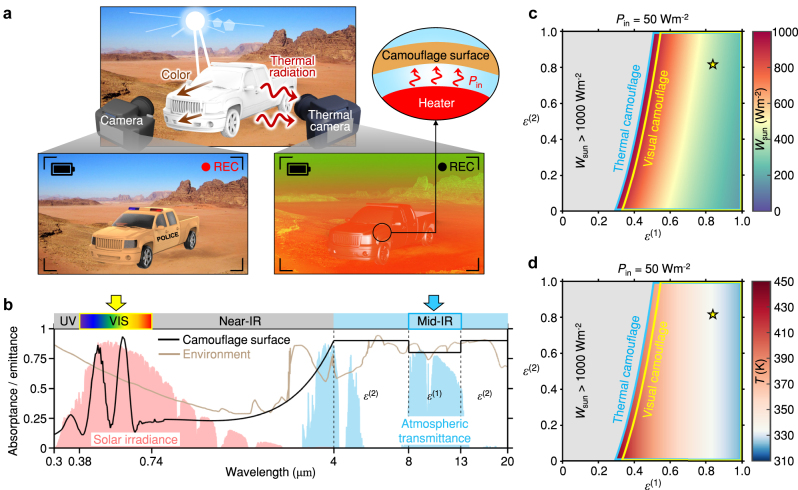
Dual-band camouflage at a single environment. (a) Schematic for visual and thermal camouflages on the terrestrial environment under internal heat expulsion. (b) Spectral absorptance (emittance) of camouflage surface and target environment. *ε* ^(1)^ is the average emittance in 8–13 µm, and *ε* ^(2)^ is the average emittance in 4–8, 13–20 µm. (c) Absorbed solar irradiance and (d) steady-state temperature of camouflage surface depending on emittances in different bands. The colored area surrounded by blue lines fulfills the thermal camouflage and steady-state conditions, and narrower regions surrounded by yellow lines can additionally fulfill the visual camouflage condition. The yellow star indicates the environmental result denoted in (b).

We first briefly review the visual and thermal camouflage conditions in a single, constant environment. In essence, camouflage is imitation of radiative signatures of surrounding environments for concealment, overcoming differences in structures and materials (Figure 1(a)). In visible wavelengths (0.38–0.78 μm), colors (including all three aspects of it—hue, colorfulness, and luminance) remain as historically and currently the most important measure for differentiating objects either by human visual perception or common imaging systems (Left schematic in Figure 1(a)) [31]. When the transmittance and radiant exitance of an object is negligible in the visible range, its color can be calculated from its spectral absorptance (which is the same as its thermal spectral emittance, *ε*(*λ*), according to Kirchhoff’s law) measured over all wavelengths (*λ*) in the visible range. Then, the criterion for visual camouflage amounts to matching the device’s color coordinates with those of the environment:

(1) $$Lab\left(\varepsilon_{\text{cam}}\right)=Lab\left(\varepsilon_{\text{env}}\right)$$

where Lab(·) is three-dimensional color coordinates in CIELAB color space [32] and *ε*_cam_ and *ε*_env_ are the spectral absorptance (or spectral emittance) of the camouflage device and environment, respectively (detailed formulations in the Methods section). On the other hand, in the mid-IR wavelength band, typical thermal imaging devices monitor the radiative heat released from objects according to Planck’s law and their emittance [33]. The most utilized band is 8–13 μm because the atmosphere is highly transparent and blackbody radiation from near-ambient-temperature (∼300 K) objects is most intense in this band, enabling long-distance surveillance with good signal-to-noise ratios [34, 35]. While another transparency window at 3–5 μm is also widely used for thermography, especially for high-temperature objects such as engines and exhaust systems, here we aim to conceal objects with only moderate or no heat generation and, thus, focus on the longer-wavelength band. Then, the thermal camouflage condition translates to matching the outgoing radiative power densities (*W*) between the camouflage device and background (Right inset in Figure 1(a)) integrated over the 8–13 μm band:

(2) $$W^{\left(8-13\;\mu m\right)}\left(\varepsilon_{\text{cam}},T_{\text{cam}}\right)=W^{\left(8-13\;\mu m\right)}\left(\varepsilon_{\text{env}},T_{\text{env}}\right)$$

where *T*_cam_ and *T*_env_ are the temperatures of the camouflage device and environment, respectively, and can be different from each other. Note that, under given environmental conditions (*ε*_env_ and *T*_env_), Eqs. (1) and (2) should be satisfied simultaneously in order to achieve dual-band camouflage, which requires a proper choice of *ε*_cam_ as a function of wavelength.

Finding such optimal *ε*_cam_, however, is not a trivial problem and the solution is not unique. While the relevant wavelength range in Eq. (1) is from 380 to 780 nm and the range in Eq. (2) is from 8 to 13 μm, completely separated from the visible wavelengths, the two equations are coupled through thermal balance conditions. For example, the temperature, *T*_cam_, in Eq. (2) depends on the emittance in the visible range as well. In fact, the rigorous calculation of the thermal balance requires the consideration of the emittance at all wavelengths over which the solar irradiance or the thermal radiation has non-negligible power, and that include wavelengths from ultraviolet up to 20 μm or longer. In addition, the camouflage device exchanges heat in non-radiative ways as well through conduction and convection. Thus, finding a solution for *ε*_cam_ requires simultaneous consideration of all these aspects. Otherwise, thermal imbalance may occur and, the camouflage device can quickly deviate from the camouflaged state.

The overall thermal balance condition can be summarized in a single equation, in a way similar to previous investigation of thermal interactions and radiative cooling in outdoor environments [36, 37]. In the model, the camouflage device can exchange heat with various surroundings such as the sun, atmosphere, and internal heater, which can be arranged in the form of net outgoing power density (*P*_cam_) that should be zero in steady state as

(3) $$\begin{array}{ll} P_{\text{cam}} & =W_{cam,all}-W_{sun,cam}-W_{atm,cam}\\ & \;-\phi_{env,cam}-P_{in,cam}=0 \end{array}$$

where *W*_cam,all_ is the radiant exitance of the camouflage device to all the others; *W*_sun,cam_ and *W*_atm,cam_ are the absorbed irradiance on the camouflage device emitted from the sun and atmosphere, respectively; *ϕ*_env,cam_ is the non-radiative heat influx density to the camouflage device from the surrounding environments; and *P*_in,cam_ is the heat flux density from a potential internal heat source such as human body to the camouflage device. The notable point in Eq. (3) is that all of its radiative terms (denoted with *W* symbols) are dependent on the spectral emittance of the camouflage device. In addition, *W*_cam,all_ term is dependent on its temperature as well, making *P*_cam_(*ε*_cam_, *T*_cam_) to be emittance and temperature dependent, similar to Eqs. (1) and (2) (detail formulations in the Methods section).

Despite this additional condition for thermal balance, the number of constraints is only three while the design space (*ε*_cam_(*λ*)) is infinite-dimensional due to the continuous nature of the wavelength. Therefore, there are infinitely many spectral emittances that satisfy all constraints for camouflage. In the following derivation, we simplify the design space in its dimensions to a bare minimum required for explanation of dual-band, dual-environment camouflage, to focus on the essence of our design principle. We divide the spectrum into the following three regions: (i) main thermal imaging window (8 μm ≤ *λ* ≤ 13 μm), (ii) the rest of the thermally relevant spectral range in the infrared (4 μm < *λ* < 8 μm, 13 μm < *λ*), and (iii) solar range (*λ* ≤ 4 μm), and assume that the emittance of the camouflage device is approximately constant within the first and second regions (denoted as $\varepsilon_{\text{cam}}^{\left(1\right)}$ and $\varepsilon_{\text{cam}}^{\left(2\right)}$ , respectively). Even with these restrictions, there are still infinitely many possible solutions due to the vast degrees of freedom in the emittance in the solar region. These solutions can result in different thermal balance and steady-state temperature for the camouflage device. Hence, we group the solutions according to their solar thermal load, *W*_sun,cam_, and use it together with $\varepsilon_{\text{cam}}^{\left(1\right)}$ and $\varepsilon_{\text{cam}}^{\left(2\right)}$ , to represent a particular group of closely related solutions.

The thermal balance problem can be solved by converting the equality constraint on color coordinates, Eq. (1), to an inequality constraint on power. Known as metamerism, there are infinite numbers of different spectral absorptance functions that produce the same color coordinates. The solar thermal load can be calculated by multiplying them with AM1.5 power spectral density and integrating them over the solar spectrum. With optimizations, one can find the lower (*W*_sun,min_) and upper (*W*_sun,max_) bounds of the absorbed solar power for given color coordinates, which determine the possible range of solar thermal load as *W*_sun,min_ ≤ *W*_sun,cam_ ≤ *W*_sun,max_ for the target color (Supplementary Note 1) [32, 38]. Then, using this inequality constraint and the equality constraints in Eqs. (2) and (3), the parameters in camouflage device (*W*_sun,cam_, $\varepsilon_{\text{cam}}^{\left(1\right)},\varepsilon_{\text{cam}}^{\left(2\right)}$ ) can be designed for the target environment. In Figure 1(b), the parameters of designed spectrum are *W*_sun,cam_ = 337 Wm^−2^, $\varepsilon_{\text{cam}}^{\left(1\right)}$ = 0.8, and $\varepsilon_{\text{cam}}^{\left(2\right)}$ = 0.9. With *P*_in_ = 50 Wm^−2^, this leads to *T*_cam_ = 341 K. Note that theses parameters are quite different from those of the target environmental parameters, *W*_sun,env_ = 495 Wm^−2^, $\varepsilon_{\text{env}}^{\left(1\right)}$ = 0.84, and $\varepsilon_{\text{env}}^{\left(2\right)}$ = 0.82 (*T*_env_ = 300 K). This is the nature of camouflage: albeit with differences in the spectral emittance and absorptance, the camouflage device can mimick the visual and thermal appearance of the environment. Mathematically this owes to the under-determined nature of the problem. There are three design variables and one temperature variable, *T*_cam_, but only two equality constraints, so the problem has a two-dimensional solution space in (*W*_sun,cam_, $\varepsilon_{\text{cam}}^{\left(1\right)},\varepsilon_{\text{cam}}^{\left(2\right)}$ ) coordinates. This indicates the possibility of multiple schemes for multi-environment active camouflage.

Before we move on to active control of the camouflage, it is useful to look more closely at the range of solutions in a single environment and their differing thermal balance conditions. Since the design space is three-dimensional and the set of all possible solutions for a particular environment condition is two-dimensional, we can plot the proper value of *W*_sun,cam_ (and the resulting *T*_cam_) as a function of $\varepsilon_{\text{cam}}^{\left(1\right)}$ and $\varepsilon_{\text{cam}}^{\left(2\right)}$ as shown in Figure 1(c) (Figure 1(d)). Note that every point on this ( $\varepsilon_{\text{cam}}^{\left(1\right)}$ , $\varepsilon_{\text{cam}}^{\left(2\right)}$ ) plane within a certain boundary is a camouflage solution for the same, given environment (*ε*_env_ and *T*_env_). The boundary (yellow lines) is determined by the inequality condition for *W*_sun,cam_ (*W*_sun,min_ ≤ *W*_sun,cam_ ≤ *W*_sun,max_), which is set by the desired color. One can observe that the possible range of ( $\varepsilon_{\text{cam}}^{\left(1\right)}$ , $\varepsilon_{\text{cam}}^{\left(2\right)}$ ) is very large. Within this region, a proper $\varepsilon_{\text{cam}}^{\left(solar\right)}$ exists for the target color; outside, the target color cannot be generated, or the thermal balance cannot be achieved. (The combined range for all colors is obtained by allowing the full range for *W*_sun,cam_ (typically 0 ≤ *W*_sun,cam_ ≤ 1000 Wm^−2^ at the ground) and bordered by blue lines in Figure 1(c) and (d), which corresponds to the ultimate allowed region for thermal camouflage.) For a common soil color of (L*, a*, b*) = (71, 4, 17), the allowed solar thermal load is found to be 162 Wm^−2^ ≤ *W*_sun,cam_ ≤ 898 Wm^−2^ (Supplementary Note 1). Since the bounded region contains all possible emittances for dual-band camouflage, it also includes a point (denoted with a star in Figure 1(c) and (d)) that corresponds to the target environment shown in Figure 1(b).

This large solution space and high degrees of freedom is beneficial in a couple of ways. First, it makes it easier to find or formulate a proper set of materials and structures for the camouflage. Second, it also allows optimizing other properties of the system without sacrificing its camouflage performance. For example, in certain applications, temperature management may be essential, such as in cooling hot engines [39] and preventing skin burns with a suit [40]. For these purposes, large $\varepsilon_{\text{cam}}^{\left(1\right)}$ design can be implemented to lower the steady-state temperature of the camouflage device, which corresponds to the bluish area in Figure 1(d). In addition, for different internal heat conditions, such as heating (*P*_in_ > 0), no heating (*P*_in_ = 0), and even cooling (*P*_in_ < 0), the possible spectral design can be deduced based on this guidance (Supplementary Note 2).

### 2.2 Dual-band camouflage in varying environments

To design dual-band camouflage for multiple environments, we first consider typical radiative features of terrestrial surfaces. Due to the myriad of geological substances and their mixtures, terrestrial background exhibits diverse colors as well classified in the Munsell chart [41]. In the IR region, particularly near the atmospheric transparency, however, their emittances are universally large and similar to one another [10, 11], which is part of the reason why IR signatures are usually a good measure of the actual temperature. To reflect these points, we use two soil spectra [42, 43] that present different colors, light and dark browns, but similarly large IR emittance above 0.8 (Supplementary Note 1).

**Figure 2:**
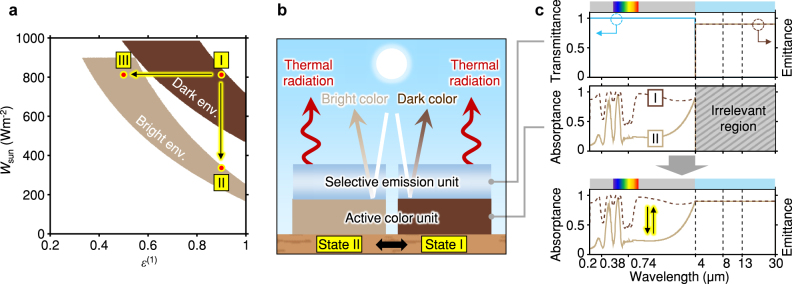
Active dual-band camouflage scheme. (a) Possible solution space for dual-band camouflage according to the target environments. The yellow arrows present the exemplary transformation between available points for dual-environmental camouflage. (b) Schematic of active dual-band camouflage device that is switchable between state I and state II. (c) Spectral properties of the selective emission unit (top), active color unit (middle), and the combined system (bottom) in active dual-band camouflage device.

Now we demonstrate how to implement dual-band camouflage in different terrestrial environments. Following our camouflage scheme explained earlier, the feasible solution space for dual-band camouflage can be obtained according to the target environments, as shown in Figure 2(a). For clear visualization, the solution space for each soil environment is plotted on ( $\varepsilon_{\text{cam}}^{\left(1\right)}$ , *W*_sun,cam_) design space, filled with their perceptual colors. Note that the solution space for each environment is two-dimensional as before, so $\varepsilon_{\text{cam}}^{\left(2\right)}$ and *T*_cam_ are uniquely determined for each point in those regions (Supplementary Note 3). Importantly, these results show that available parametric spaces of bright and dark environments are apart, confirming that some change of the spectral properties of the camouflage system is necessary for this dual-band dual-environmental camouflage to work. We consider two distinct transformations between two solution spaces with dark and light brown colors: (i) changing *W*_sun,cam_ and holding $\varepsilon_{\text{cam}}^{\left(1\right)}$ constant (state I ↔ state II); or (ii) holding *W*_sun,cam_ constant and changing $\varepsilon_{\text{cam}}^{\left(1\right)}$ (state I ↔ state III). In both cases, $\varepsilon_{\text{cam}}^{\left(2\right)}$ can be designed freely, but to lower design complexity, we consider $\varepsilon_{\text{cam}}^{\left(2\right)}$ to be the same as $\varepsilon_{\text{cam}}^{\left(1\right)}$ . Then, the spectral change can be arranged as follows: case (i) changes the emittance only in the solar range, and case (ii) changes the emittance at all wavelengths (Supplementary Note 3). Thus, here we address the design-friendly case (i) requiring the change of limited parameters, i.e., the color and *W*_sun,cam_. In addition, among various combinations of state I and state II belonging to case (i), we implement a system with high $\varepsilon_{\text{cam}}^{\left(1\right)}$ and $\varepsilon_{\text{cam}}^{\left(2\right)}$ , which can lower the temperature of the camouflage device with radiative cooling effect.

### 2.3 Design of active dual-band camouflage device

For the spectral-selective switching operation of the case (i), we propose a camouflage design composed of a thermally emissive layer and a color-changing layer (Figure 2(b)). The top thermal emitter is transparent in the solar region and is highly emissive above 4 μm wavelengths. Through the transparency in the solar band at the top, the active color unit at the bottom can control the system’s apparent colors according to the camouflage states. On the other hand, the spectral properties of the active color unit over 4 μm wavelengths do not affect the observed thermal properties of the combined system because the thermally emissive top layer blocks the radiation from or to the color unit. This aspect simplifies the design and material choices. Based on this configuration, the integrated device can present the spectral-selective switchable emittance, as shown in Figure 2(c).

**Figure 3:**
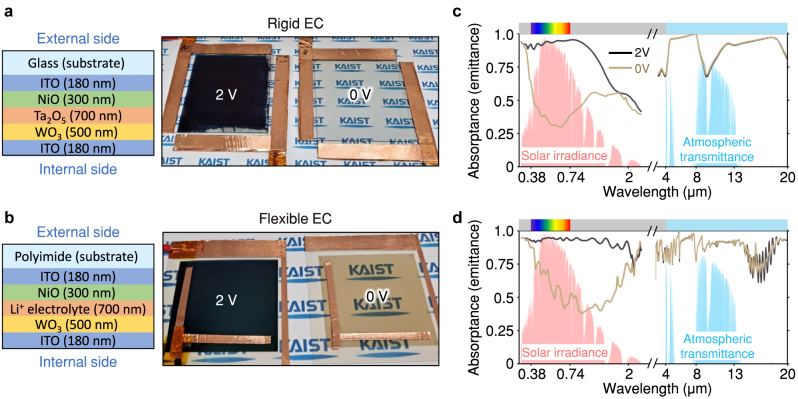
Designed electrochromic camouflage devices. Schematics and photographic images for (a) rigid and (b) flexible electrochromic devices, and their spectral absorptance (emittance) depending on applied voltage for the (c) rigid and (d) flexible devices.

Following the above scheme, we experimentally design the active dual-band camouflage devices by stacking electrochromic layers and highly emissive substrate, corresponding to the active color and selective-emission units, respectively (Figure 3(a) and (b)). For versatility of the design, we chose to make the device nearly transparent when the voltage is not applied so that the natural color of the device can be easily modified by placing a colored surface underneath the device. The color of the device when the voltage is applied can be varied by choosing the electrochromic material or the applied voltage. Hence, visibly transparent materials are selected for the electrochromic units, including conductors (indium tin oxide; ITO), ion storage (NiO), and electrolytes (Ta_2_O_5_ or Li-based polymeric gel). We chose tungsten oxide (WO_3_) as the electrochromic layer material for its good visible contrast between the transparent and opaque states. These configurations are widely used for the on-off operation of transparency [44, 45] and are reliable enough to be commercialized for passenger aircraft windows. In addition, since its coloration is based on reversible ion transfer, the device can be bleached back to the original transparent state with the opposite electrical bias. For the transparent substrates, we adopted glass and polyimide, considering different potential applications that would require rigidity or flexibility, respectively. The choice of the electrolyte (Ta_2_O_5_ or polymeric gel) is also based on the consideration of this differing target mechanical property. The details of the fabrication process are covered in the Method section.

The photographic images show the significant transition of colors depending on the applied voltage, where 0 V sets transparency in the rigid case or light colors in the flexible case, while 2 V sets near black in all cases. To examine the spectral change, the designed samples were measured by UV–vis spectrometer and FT-IR spectrometer. The results in Figure 3(c) and (d) present that the solar absorption substantially increases with voltage, particularly in the visible region, which indicates the change of solar thermal load from 428 (541) Wm^−2^ to 873 (920) Wm^−2^ for the rigid (flexible) case under air mass (AM) 1.5 global standard spectrum. On the other hand, for the substrate side, the emittance over 4 μm wavelengths remains high above 0.8 regardless of applied voltage.

To evaluate the camouflage performances, we designed four virtual terrestrial environments based on ink-jet printing, which displays various colors with high IR emittances like actual environments (Supplementary Note 4). The virtual environments of A, B, C, and D represent the colors of snow, deep blue silt, bright sand, and black soil, whose labels in the Munsell chart correspond to 5 PB/6/1, 5 PB/2.5/1, 10R/5/3, and N2.5, respectively. Also, in order of type, their solar thermal loads are 389 Wm^−2^, 884 Wm^−2^, 557 Wm^−2^, and 932 Wm^−2^, respectively. For these target environments, the possible solution spaces for dual-band camouflage were calculated and compared with the designed camouflage devices (Supplementary Note 5). The results illustrate that all the states of camouflage devices belong to the solution space of each environment as: rigid-0V, rigid-2V, flexible-0V, and flexible-2V are included in the solution space of the environment A, B, C, and D, respectively. In addition, the switching operations of rigid and flexible devices adhere to case (i). Therefore, from these theoretical results, one can expect that the rigid (flexible) device can actively camouflage in environments A and B (C and D).

### 2.4 Outdoor measurements

**Figure 4:**
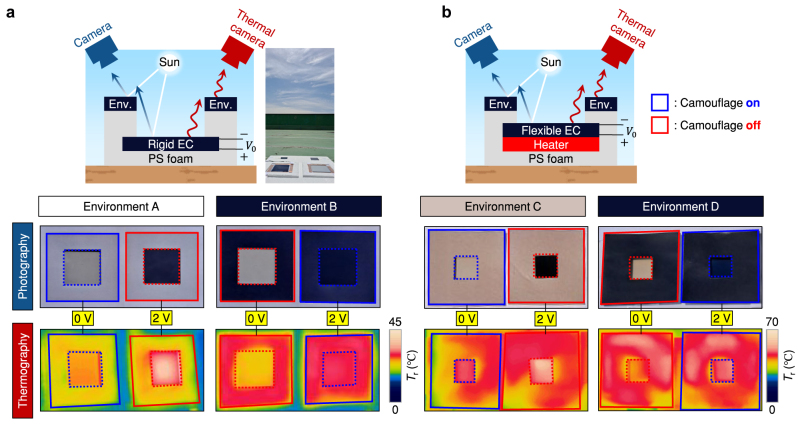
Experimental results for dual-band dual-environmental camouflage. Schematic, photographic image, and thermographic image for (a) rigid and (b) flexible camouflage devices. The internal resistive heater in (b) releases ∼50 Wm^−2^ power density.

Now we illustrate the outdoor experimental results for the designed active dual-band camouflage devices. To determine whether the device is recognizable against the environment, the environmental sample is placed to surround the camouflage device while preventing conductive heat flow between them for thermal non-equilibrium. The outdoor results show that the rigid sample is visually thermally camouflaged to environment A for 0 V and environment B for 2 V (Figure 4(a)). On the other hand, for the opposite voltage applied, the camouflage device is clearly distinguished from the environmental samples, which implies two essential points: first, both samples are thermally separated well as intended; secondly, without switching operation, the camouflage function in either band will be easily neutralized at different environment. In flexible cases, the resistive heater is implemented under the camouflage devices to set the internal heat of *P*_in,cam_ = 50 Wm^−2^, which corresponds to heat dissipation of the human body [46, 47]. As expected, the flexible sample shows excellent visual camouflage performance to environment C for 0 V and environment D for 2 V (Figure 4(b)). For the thermal vision, although windy weather results in a non-uniform temperature distribution across the environmental samples (which will be very common in real situations), the camouflage device in proper state reproduces the thermal signature of target environments. Whereas in the improper state, the thermal signature of the camouflage device does not match any region of the environmental samples (Supplementary Note 6). Also, from the theoretical results in Supplementary Note 5, one can check that the possible solution space includes the on-off states of designed camouflage devices when *P*_in,cam_ is small enough.

To examine radiative cooling of camouflage device – the useful functionality of high-emissive design, we conducted additional outdoor experiment (Supplementary Note 7). For comparison, non-cooling samples are designed to have much lower emittance, but similar solar thermal load compared to camouflage devices. The results show that the camouflage devices are substantially cooler than reference samples with temperature difference of 6.6 K and 8.1 K for rigid-0V and rigid-2V, respectively. Therefore, our camouflage scheme with high emittance can be applied for applications requiring temperature regulation.

While the steady-state model in Eq. (3) provides useful insights into camouflage, the application of camouflage in much more dynamic environments requires additional considerations due to the transient nature of both the environment and the camouflage system. In such cases, a combination of steady-state and transient models would be required to comprehensively address the complexity of problem. Realization of such dynamic camouflages would require incorporation of additional tuning mechanism as well, such as phase change materials [27] and graphene [26] for emissivity control. Thermoelectric elements [16] may also be useful as a rapid temperature modulation mechanism in dynamic environments.

## 3 Conclusions

In conclusion, we theoretically derived possible spectral designs for visual and thermal camouflage on two different terrestrial environments, and experimentally realized them based on high-emissive electrochromic devices. Particularly, we showed that the proposed design is feasible for terrestrial dual-environmental camouflage, allowing simple transformation via change of the spectral emittance in the visible range. Based on this theoretical guidance, we expect that, without laborious optimization and trial-and-error methods, more feasible solution for the camouflage can be found. Furthermore, besides camouflage functions, additional functionality, such as radiative cooling, flexibility, compatibility with existing camouflage scheme, can be achieved due to its large design degrees of freedom, which will be beneficial for applications in security and defense.

## 4 Methods

### 4.1 Calculation of the camouflage and steady-state conditions

To solve the Eqs. (1)–(3), we enumerate the formulas in detail. First, for non-photoluminescent object, the perceived color can be quantified in CIE XYZ color space as

(4) $$\vec{X}=\frac{100}{N}\bar{x}DR$$

where $\vec{X}$ is the color coordinate, also referred to as tristimulus values (3 × 1 vector); $\bar{x}$ is the color matching functions of human’s photoreceptor (3 × *k* matrix in which the row represents color matching functions of $\bar{x}$ , $\bar{y}$ , and $\bar{z}$ , and the column represents the number of wavelength points); *D* is the illuminant (*k* × *k* diagonal matrix); *R* is the reflectance of target object (*k* × 1 vector); *N* is $\bar{y}DJ_{k,1}$ where *J*_*k*,1_ is a *k* × 1 all-ones vector. When the transmittance is negligible, the Eq. (4) can be arranged with the emittance matrix (*E*: *k* × 1 vector) as $\vec{X}=\frac{100}{N}\bar{x}D\left(1-E\right)$ . For perceptual uniformity of the color space, $\vec{X}$ can be transformed into the coordinate in CIE LAB color space based on the well-known transformation method [32], resulting in each term of Eq. (1).

Since the Eq. (3) encompasses the radiative heat transfer of Eq. (2), we first denote the terms in Eq. (3) following the convention of previous work [48] as

(5a) $$\begin{array}{ll} W_{cam,all}\left(T_{\text{cam}}\right) & =\iint {\tilde{I}}_{\text{BB}}\left(\lambda ,T_{\text{cam}}\right)\varepsilon_{\text{cam}}\left(\lambda ,\Omega ,T_{\text{cam}}\right)\cos\theta d\lambda d\Omega \end{array}$$

(5b) $$\begin{array}{ll} W_{sun,cam}\left(T_{\text{cam}}\right) & =\iint {\tilde{I}}_{\text{sun}}\left(\lambda ,\Omega \right)\varepsilon_{\text{cam}}\left(\lambda ,\Omega ,T_{\text{cam}}\right)\cos\theta d\lambda d\Omega \end{array}$$

(5c) $$\begin{array}{ll} W_{atm,cam}\left(T_{\text{cam}},\;T_{\text{amb}}\right) & =\iint {\tilde{I}}_{\text{atm}}\left(\lambda ,\Omega ,T_{\text{amb}}\right)\\ & \;\times \varepsilon_{\text{cam}}\left(\lambda ,\Omega ,T_{\text{cam}}\right)\cos\theta d\lambda d\Omega \end{array}$$

(5d) $$\phi_{cam,env}\left(T_{\text{cam}},T_{\text{env}}\right)=h_{\text{c}}\left(T_{\text{cam}}-T_{\text{env}}\right)$$

where $\int \left(\;\cdot \;\right)d\Omega =\int_{0}^{2\pi }\int_{0}^{\pi /2}\left(\;\cdot \;\right)\;\sin\theta \;d\theta d\phi$ is the hemispherical integration for solid angle Ω or two angular coordinates of azimuthal angle *ϕ*, and zenith angle *θ*; ${\tilde{I}}_{\text{BB}}$ , ${\tilde{I}}_{\text{sun}}$ , and ${\tilde{I}}_{\text{atm}}$ (Wm^−2^ μm^−1^ sr^−1^) are the spectral radiances of a blackbody, the sun, and the atmosphere; *λ* is the wavelength; *T*_amb_ is the ambient temperature; *h*_c_ (Wm^−2^ K^−1^) is the effective non-radiative heat transfer coefficient. By limiting the spectral range of Eq. (5a) to 8–13 μm, the outgoing radiative power densities of Eq. (2) can be obtained. In this work, ${\tilde{I}}_{\text{sun}}$ corresponds to global standard AM1.5 condition, and ${\tilde{I}}_{\text{atm}}$ is calculated by using the atmospheric transparency in mid-latitude (MODTRAN6 [49]). Also, the rest of parameters are set as previous work [48]: *T*_amb_ = *T*_amb_ = 300 K, and *h*_c_ = 6 Wm^−2^ K^−1^.

### 4.2 Fabrication of the electrochromic devices

The NiO thin films were deposited on the indium tin oxide (ITO)-coated glass substrate (sheet resistance ≈ 10 Ω/square, 5 × 5 cm^2^) by the reactive DC/RF magnetron sputtering with a 4-inch-diameter Ni target. Sputtering was performed with Ar/O_2_ gas flow (purity ≈ 99.998 %, 90/10 sccm) at a constant working pressure of 4 × 10^−3^ Torr and fixed DC output power of 500 W to deposit NiO films (thickness ≈ 300 nm). Before every deposition of thin films, a pre-sputtering process was performed with Ar atmosphere for 10 min. To fabricate a monolithic inorganic electrochromic device, Ta_2_O_5_, WO_3,_ and ITO were sequentially deposited on the NiO/ITO/Glass substrate using a sputtering process. The Ta_2_O_5_ film (thickness ≈ 700 nm), as an ion conductor layer, was deposited with a Ta_2_O_5_ target under Ar/O_2_ gas flow (purity ≈ 99.998 %, 99/1 sccm) and at a constant radio frequency (RF) output power of 500 W. The WO_3_ film (thickness ≈ 500 nm), as the electrochromic layer, was deposited with a tungsten target under Ar/O_2_ gas flow(purity ≈ 99.998 %, 75/25 sccm) and at fixed DC output power of 500 W. The ITO thin film (thickness ≈ 180 nm), as a transparent conducting oxide, was deposited under Ar/O_2_ gas flow (purity ≈ 99.998 %, 40/1 sccm) and at continuous output power of 500 W for electrochromic devices [50].

The flexible electrochromic devices were fabricated via reactive DC magnetron sputtering at room temperature. The NiO thin films (thickness ≈ 300 nm) were deposited on the ITO-coated polyimide (PI) (sheet resistance ≈ 28 Ω/square, 5 × 5 cm^2^) substrate with the Ni target under Ar/O_2_ gas flow (purity ≈ 99.998 %, 90/10 sccm) at a constant working pressure of 4 × 10^−3^ Torr and fixed DC output power of 500 W. The Li-based polymeric electrolyte (soulbrain, SWOPE; thickness ≈ 700 nm) was coated on the NiO/ITO/PI films by the bar-coating process and cured via the ultraviolet exposer (UV LED lap dryer 150, Phoseon Technology). Finally, the WO_3_ (thickness ≈ 500 nm) and ITO (thickness ≈ 180 nm) layers were deposited on the multi-layered films under the same deposition parameters of the rigid type to fabricate flexible electrochromic devices and measure properties. The prepared NiO/ITO/PI films and WO_3_/ITO/PI films, under the same deposition parameters, were laminated with the Li-based polymeric gel electrolyte (soulbrain, SWOPE; thickness ≈ 700 nm) and the integrated thin films were cured by the ultraviolet exposer (UV LED lap dryer 150, Phoseon Technology) for flexible electrochromic devices [51] (Supplementary Note 8).

### 4.3 Outdoor experimental conditions

For visible and thermal imaging, we utilized a camera (Samsung A51) and a thermal camera (FLIR E75 sensing 8–14 μm). The outdoor measurements were conducted at KAIST in Daejeon, South Korea, and the climate conditions are as follows: for rigid (flexible) case, the averaged solar irradiance was 667 Wm^−2^ (1000 Wm^−2^), the averaged wind speed was 1.3 ms^−1^ (1.0 ms^−1^), the averaged humidity was 53 % (62 %), and the sky condition was cloudless, from 11:00 to 13:00. (Supplementary Note 7).

## Supporting information

Supporting Information is available from the Wiley Online Library or from the author.

## Supplementary Material

Supplementary Material Details
