# Supplementary material for: Visual and thermal camouflage on different terrestrial environments based on electrochromism
Source: Nanophotonics. 2023 Jun 12;12(15):3199–209. doi: 10.1515/nanoph-2023-0244 (PMC11501304; doi:10.1515/nanoph-2023-0244)
Supplement: Supplementary file 1 — Supplementary Material Details [file j_nanoph-2023-0244_suppl_001.pdf]

Supplementary material

Visual and thermal camouflage on different terrestrial environments based on electrochromism

Suwan Jeon[1,2], Su Eon Lee[3*], Wonjoong Kim[4*], Sun Hee Lee[5], Seokhwan Min[1], Seung Won Seon[6], Seung Ho Han[5], Bong Hoon Kim[3†], Heon Lee[4†], Jonghwa Shin[1†]*

[1]Department of Materials Science and Engineering, KAIST, Daejeon 34141, Republic of Korea

[2]Department of Nano Mechanics, Korea Institute of Machinery & Materials (KIMM), Daejeon, 34103, Republic of Korea

[3]Department of Robotics and Mechatronics Engineering, DGIST, Daegu 42988, Republic of Korea

[4]Department of Materials Science and Engineering, Korea University, Seoul 02841, Republic of Korea

[5]Electronic Convergence Materials & Device Research Center, KETI, Seongnam 13509, Republic of Korea

[6]Department of Smart Wearable Engineering, Soongsil University, Seoul 06978, Republic of Korea

Note S1: Range of solar thermal load for target color

Note S2: Extended data for dual-band single-environmental camouflage

Note S3: Extended data for dual-band dual-environmental camouflage

Note S4: Spectral properties of virtual terrestrial samples

Note S5: Validity check for camouflage of designed samples

Note S6: Reliability check for outdoor measurement results

Note S7: Additional outdoor measurement and climate conditions

Note S8: Schematic for fabrication process

Note S1: Range of solar thermal load for target color

We demonstrate the possible range of solar thermal load for target colors based on metamerism. Due to one-to-many relations between color and spectral reflectance (or absorptance in opaque material), the absorbed solar power can be ranged broadly [1]. To examine the upper and lower bounds of solar thermal load, we exploit the metamer search algorithm developed in our previous work [2]. As target colors, we use experimental data for soils #1–6 and soils #7–9, obtained from Ref. 3 and Ref. 4, respectively (Soil #1: Andisol C = 5%, Soil #2: Andisol C = 19%, Soil #3: Inceptisol C = 49%, Soil #4: Mollisol C = 4%, Soil #5: Oxisol C = 1%, Soil #6: Oxisol C = 3%, Soil #7: selected soil 1, Soil #8: selected soil 2, Soil #9: selected soil 3). The soil #5 and soil #9 correspond to dark-colored and bright-colored environments in the manuscript. The spectral properties of soils in Fig. S1(a) show that the absorptive profiles in solar band are variant but the emittance above 4 um are commonly large (which is very essential terrestrial condition in this work). Figure S1(b) presents the solar thermal loads of soil spectra (colored markers) and the possible ranges to express the target colors (colored bars). These results indicate that the camouflage surface can be visually camouflaged as generating the target color (Fig. S1(c)) while absorbing sunlight over a fairly free range.

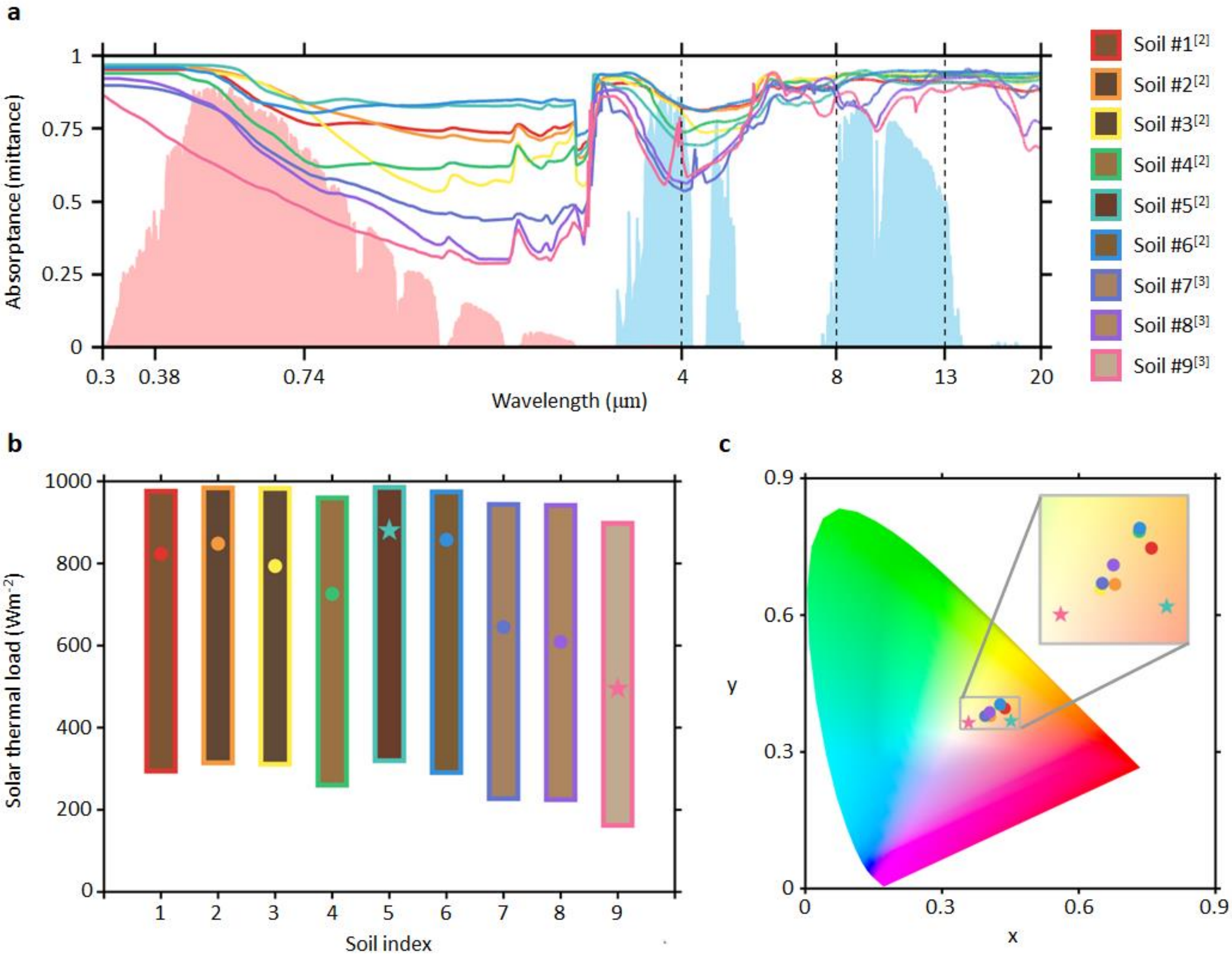

Figure S1. Radiative properties of soils. (**a**) Spectral absorptance (emittance), (**b**) the possible range of solar thermal load, and (**c**) the color coordinates in CIE XYZ color space, depending on the soil type. The solar thermal loads of soil #1–9 correspond to the marker in (b). The x and y ranges of inset in (c) are 0.34–0.47, and 0.35–0.42, respectively.

Note S2: Extended data for dual-band single-environmental camouflage

We show the available design regions for dual-band camouflage when the concealed object releases heat (P_{in} = 0, 200, 400 $\mathrm{Wm^{-2}}$) into the camouflage surface. For the bright-colored (soil #9 in Fig. S1) and dark-colored (soil #5 in Fig. S1) environment, the visual and thermal camouflage regions are shifted to the lower $\varepsilon^{(1)}$ as P_{in} increases (Figs. S2, S3); because the higher steady-state temperature caused by internal heat requires the lower emissivity to accord with the radiative intensity of the environment. Another notable point is that the target environment (marked in yellow star) can be out of the camouflage region for large P_{in}, implying that decoration with environmental materials (which is widely used in military fields) are not feasible for dual-band camouflage purpose. Therefore, based on the guidance of this work, the camouflage devices must be carefully designed in terrestrial regime particularly when P_{in} is non-zero.

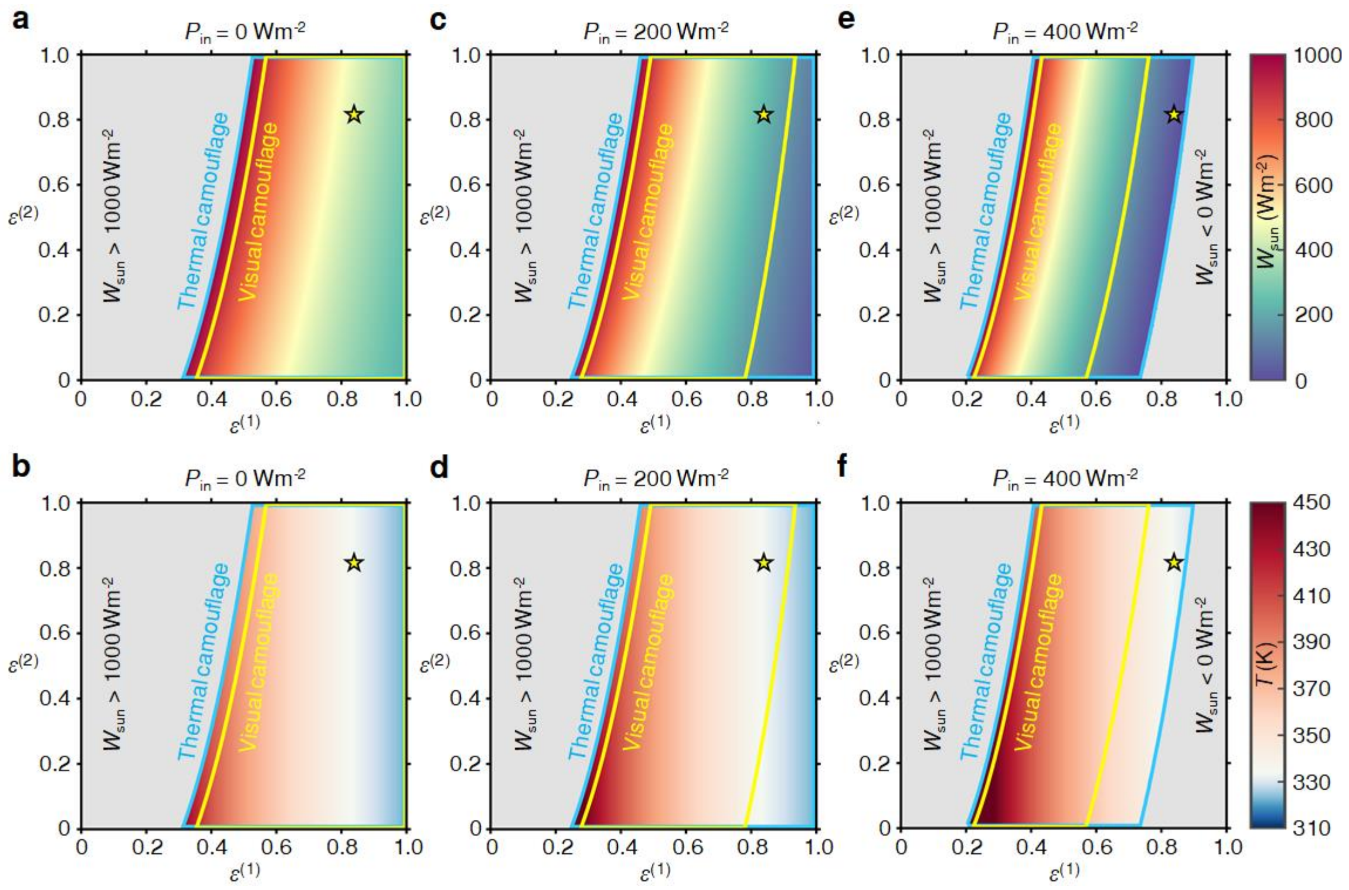

Figure S2. Dual-band camouflage for bright-colored environment. (**a**, **c**, **e**) Absorbed solar irradiances and (**b**, **d**, **f**) steady-state temperature of camouflage surface depending on emittances in different bands.

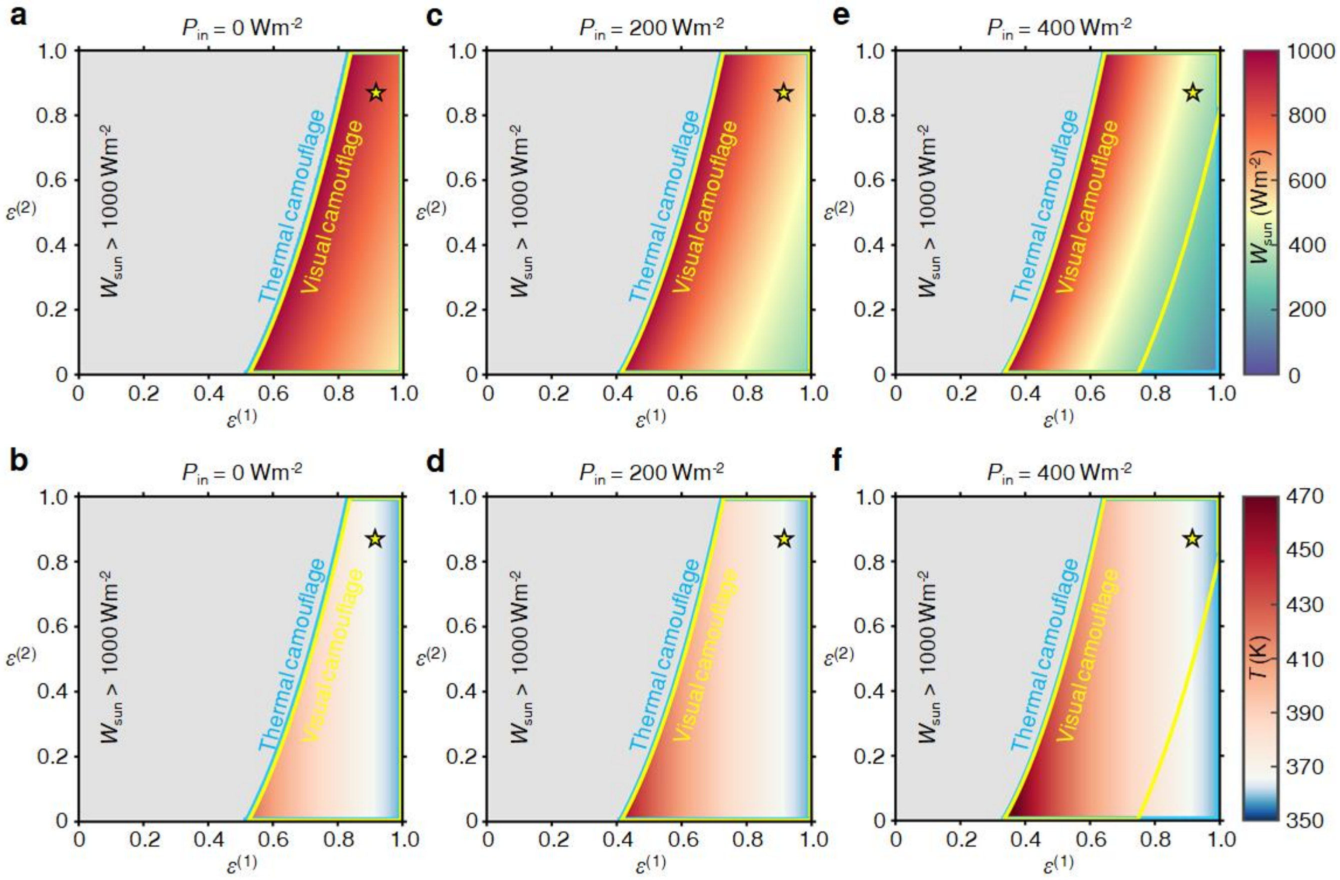

Figure S3. Dual-band camouflage for dark-colored environment. (**a**, **c**, **e**) Absorbed solar irradiances and (**b**, **d**, **f**) steady-state temperature of camouflage surface depending on emittances in different bands.

Note S3: Extended data for dual-band dual-environmental camouflage

We provide the other essential parameters in Fig. 2(a) to achieve dual-band camouflage at different environments. Figures S4(a, b) illustrate the steady-state temperature and $\varepsilon^{(2)}$ at possible dual-band camouflage conditions according to the target environments. The camouflage states of I, II, and III are the particular case of $\varepsilon^{(1)} = \varepsilon^{(2)}$ that corresponds to the black solid lines in Fig. S4(b). For these representative states that specify the spectral properties of $\varepsilon^{(1)}$, $\varepsilon^{(2)}$, and W_{sun}, we obtained the corresponding spectra in Figs. S4(c, d). As noted in the manuscript, the transition between I and III requires the change of absorptive spectra in overall wavelengths unlike the transition between I and II.

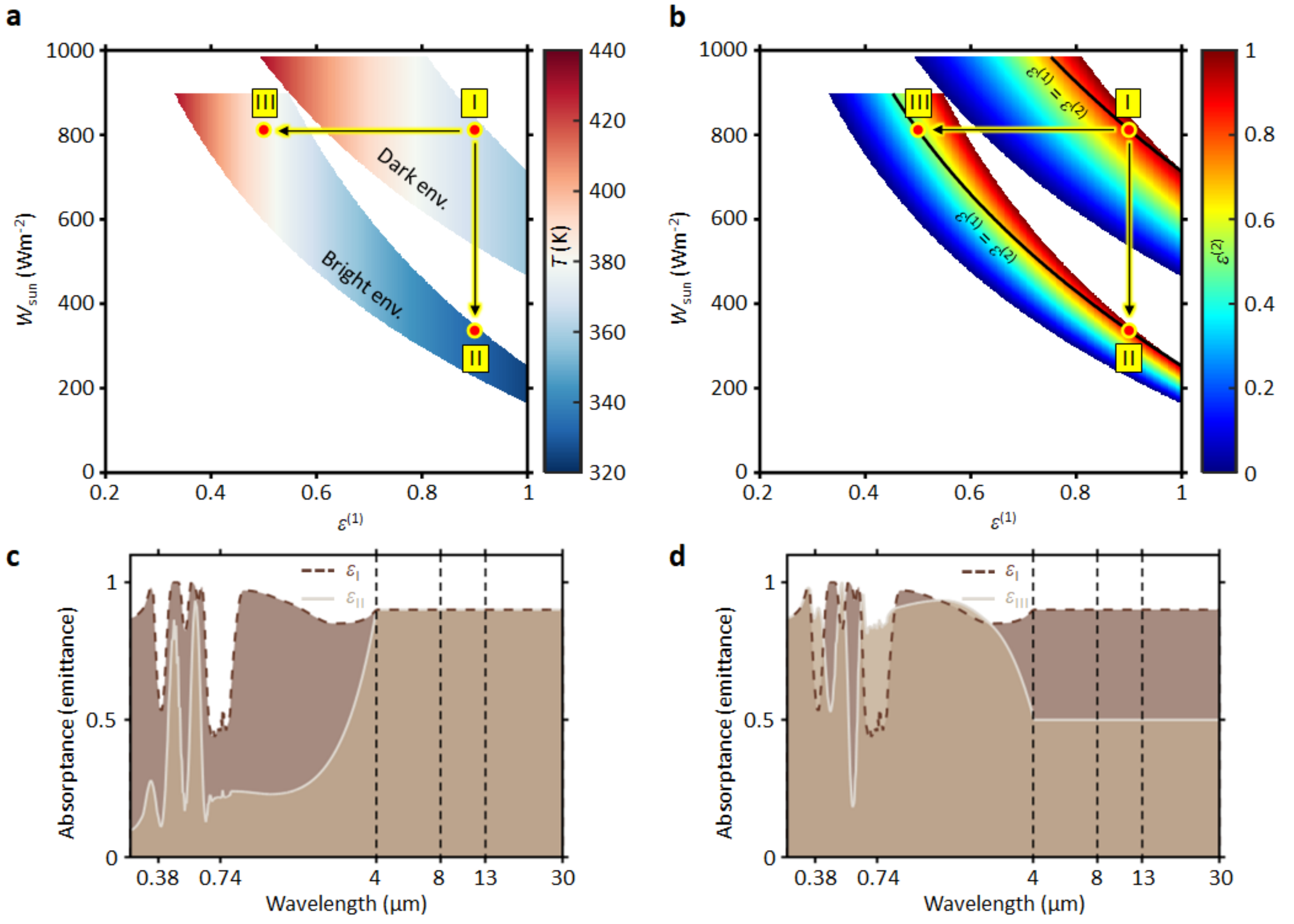

Figure S4. Dual-band dual-environmental camouflage. (**a**) Steady-state temperature and (**b**) 2nd band emittance in dual-band camouflage regions for bright-colored and dark-colored environments. The black

line in (b) accords with the condition of $\varepsilon^{(1)} = \varepsilon^{(2)}$ where three representative states (I, II, and III) are assigned. (**c**, **d**) Spectral absorptance (emittance) depending on camouflage states.

Note S4: Spectral properties of virtual terrestrial samples

Figure S5 shows the spectral absorptance (emittance) of virtual terrestrial samples measured by UV-VIS spectrometer and FT-IR spectrometer. From these results, one can check that the designed samples reproduce the essential properties of the actual terrestrial environment in Fig. S1, which are various spectral profiles in solar band and commonly large emittance in mid-IR range [5-6].

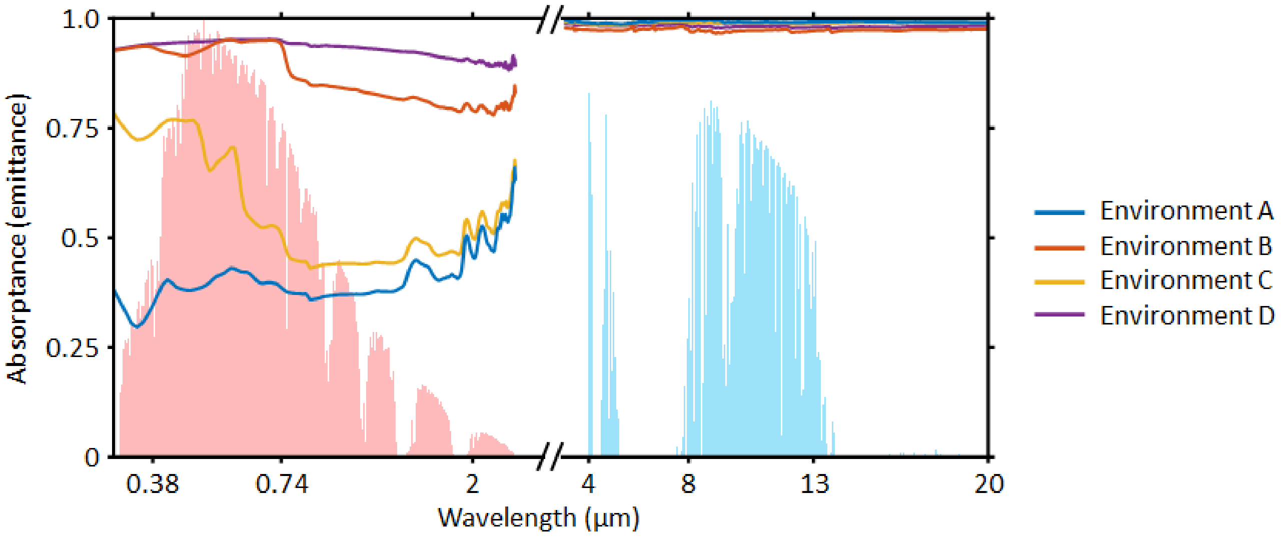

Figure S5. Spectral absorptance (emittance) of ground-like environments.

Note S5: Validity check for camouflage of designed samples

We identify whether the designed devices can actively camouflage on different virtual environments as following the conversion scheme, which is to maintain $\varepsilon^{(1)}$ while varying W_{sun}. For the measured spectra in Fig. S5, all the possible camouflage states at the virtual environments can be calculated as shown in Fig. S6. As expected, the spectral properties of designed camouflage devices (red dots) belong to the target area of each sample (colored areas). More specifically, Table S1 shows CIEDE2000 color distances [7] (ΔE_{00}) between camouflage devices and virtual environments where the diagonal color distances are small enough to make color difference difficult to perceive unlike other off-diagonal cases. Also, Table S2 presents that the released radiative heat from the camouflage device is similar with the radiative heat from the target virtual environment.

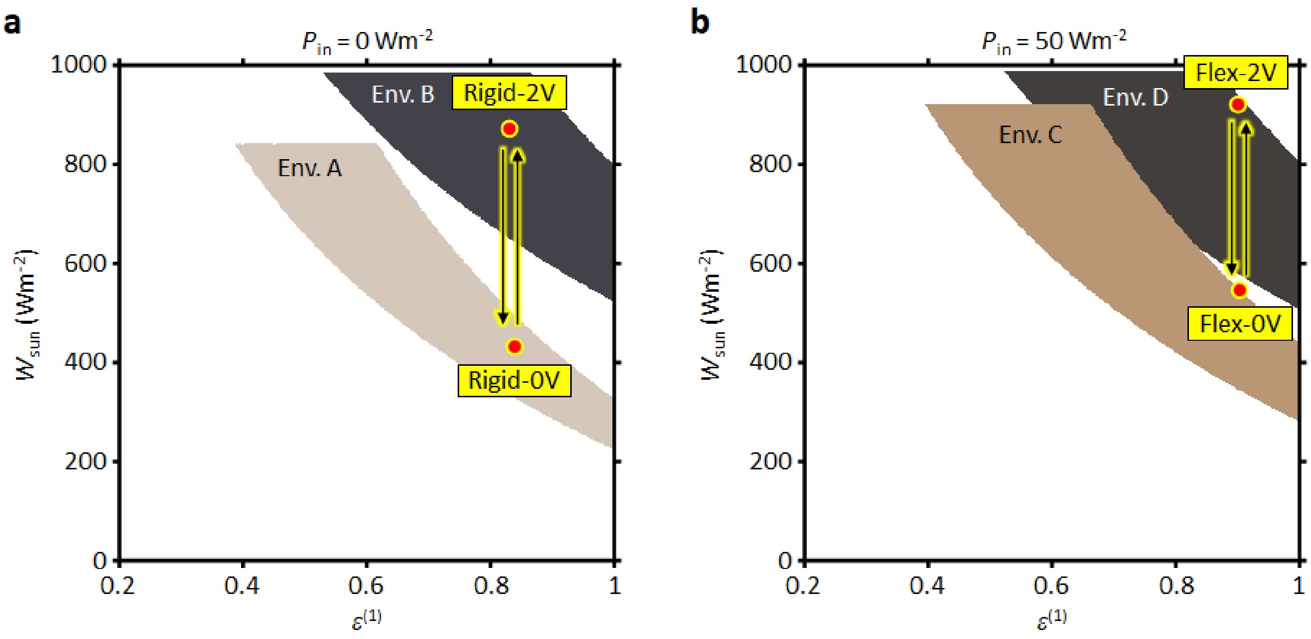

Figure S6. Active camouflage scheme of designed samples. (**a**, **b**) Dual-band camouflage regions for ground-like environment A, environment B, environment C, and environment D. The red dots present the conditions of designed camouflage devices.

Table S1. Color distance (ΔE_{00}) between camouflage device and environment.

	Rigid-0V	Rigid-2V	Flex-0V	Flex-2V
Env. A	7.4	49.6	12.9	50.3
Env. B	56.5	4.6	49.2	5.8
Env. C	16.6	38.8	8.3	38.2
Env. D	56.0	4.8	47.0	6.2

Table S2. Radiative heat release and steady-state temperature.

	Radiative heat release (Wm^{-2})	Steady-state temperature (K)
Env. A	215.1	326.5
Env. B	320.6	360.8
Env. C	250.7	338.5
Env. D	333.5	363.4
Rigid-0V	194.9	331.5
Rigid-2V	281.2	362.9
Flex-0V	243.9	343.4
Flex-2V	325.6	368.9

Note S6: Reliability check for outdoor measurement results

We reexamine the thermal camouflage performance of flexible device for the outdoor measurement. At the measurement day near noontime (Fig. S9(a)), the wind caused non-uniform temperature distribution of the virtual environmental samples (Fig. 4(b)), which makes it difficult to quantify the thermal camouflage of the designed device. So, we divide the area of the virtual environment to compare their signatures with that of camouflage device located in the center. Figure S7 and S8 shows how much radiative heat from the virtual environment is smaller (blue) or larger (red) than that of the flexible camouflage device. Compared to the environment C, the flexible-0V shows minor difference of radiative signature down to 0% and 4% (without the sign), which is smaller than 6% and 6% of flexible-2V for P_{in} = 0, 50 Wm^{-2}, respectively (Fig. S7(a)). Also, compared to the environment D, flexible-2V presents lower difference of radiative signature down to 0% and 1% (without the sign) than flexible-0V of 3% and 11% (Fig. S7(b)). Therefore, the flexible-0 and flexible-2V can camouflage on the environment C and environment D, respectively.

Figure S9 shows the photographic images for the outdoor measurement setup that includes power supply, resistive heater, data logger, camouflage devices, virtual environments, and reference samples (described in note S7).

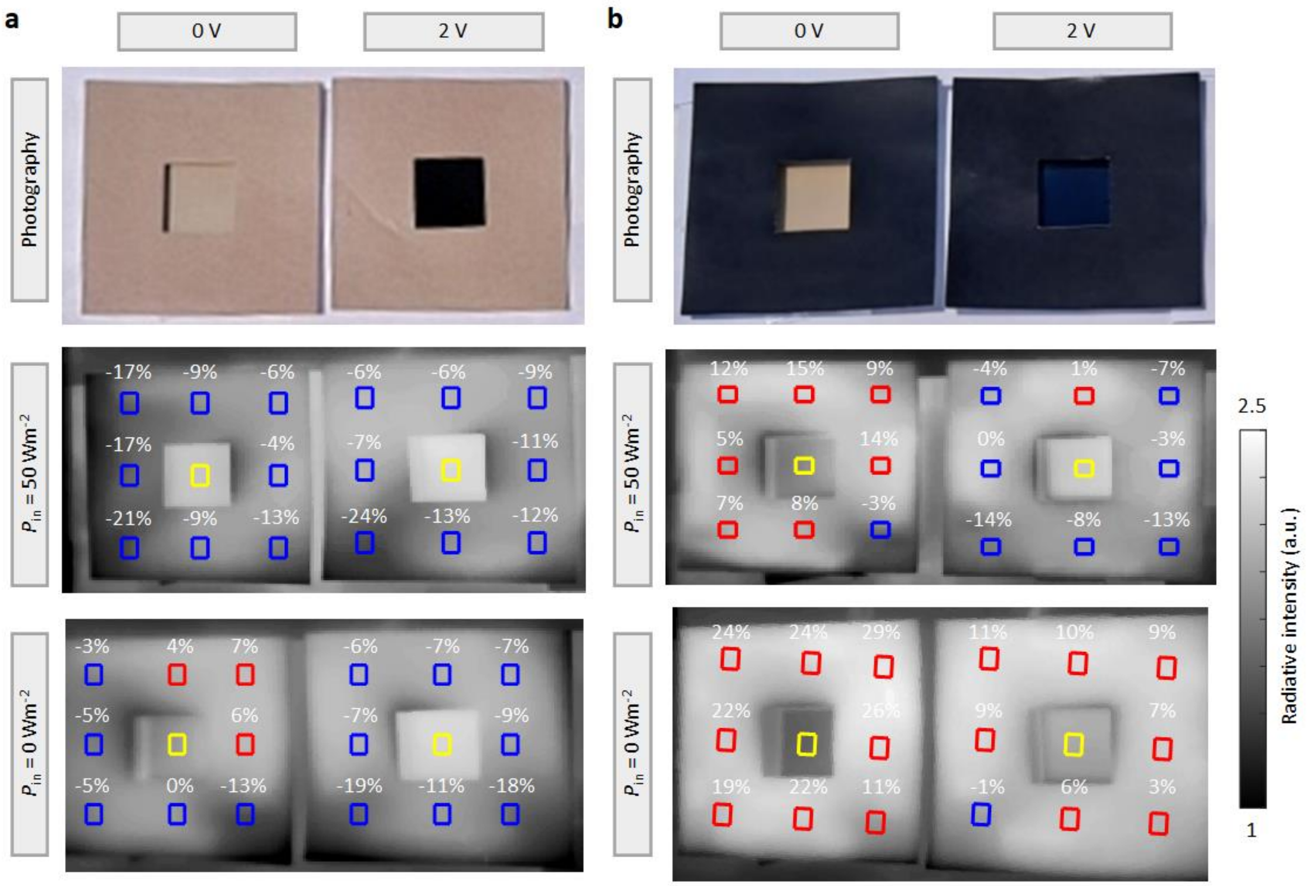

Figure S7. Camouflage performance of flexible sample. The photographic (top) and thermographic (middle and bottom) images at (**a**) environment C and (**b**) environment D. The area encircled by blue and red lines are cooler and hotter than the camouflage surface colored in yellow.

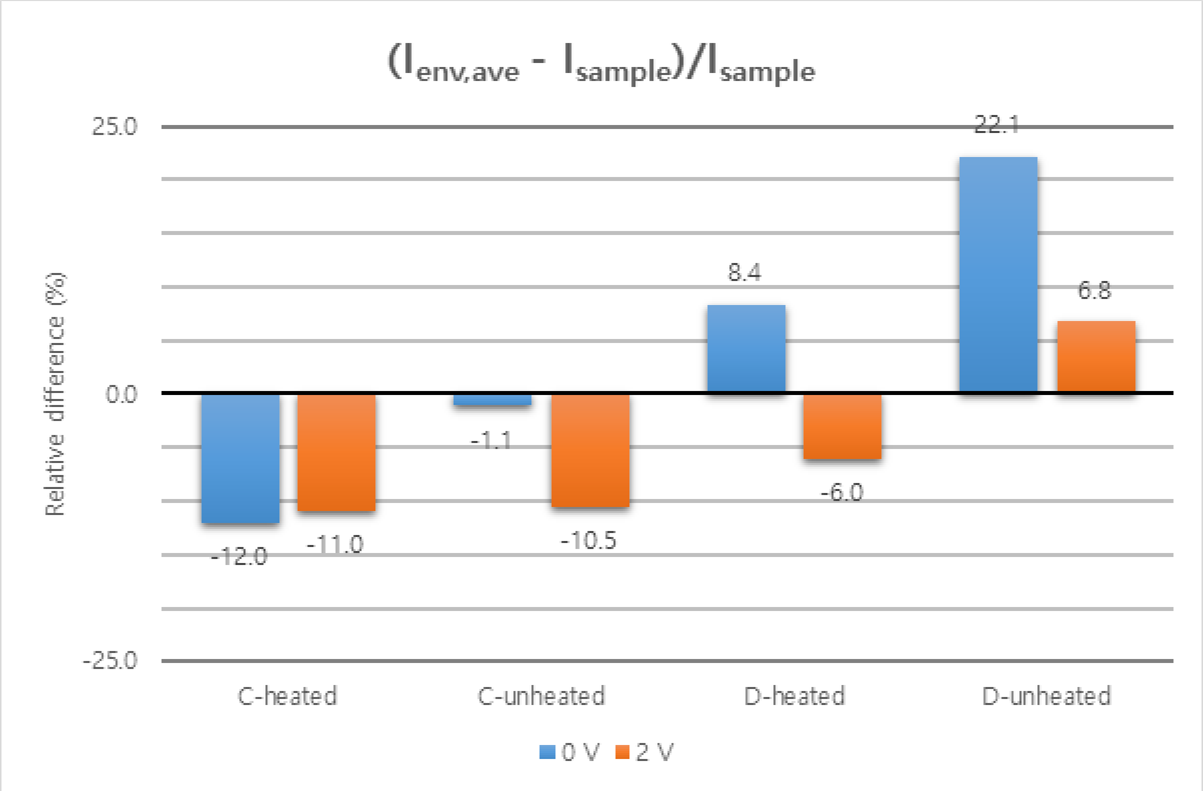

Figure S8. Camouflage performance of flexible samples. These data summarize Fig. S7 (averaged relative difference of the radiation intensity in the environment compared to the sample area) for easy comparison. Note that the sample is designed to blend with Environment C at 0 V and with Environment D at 2 V.

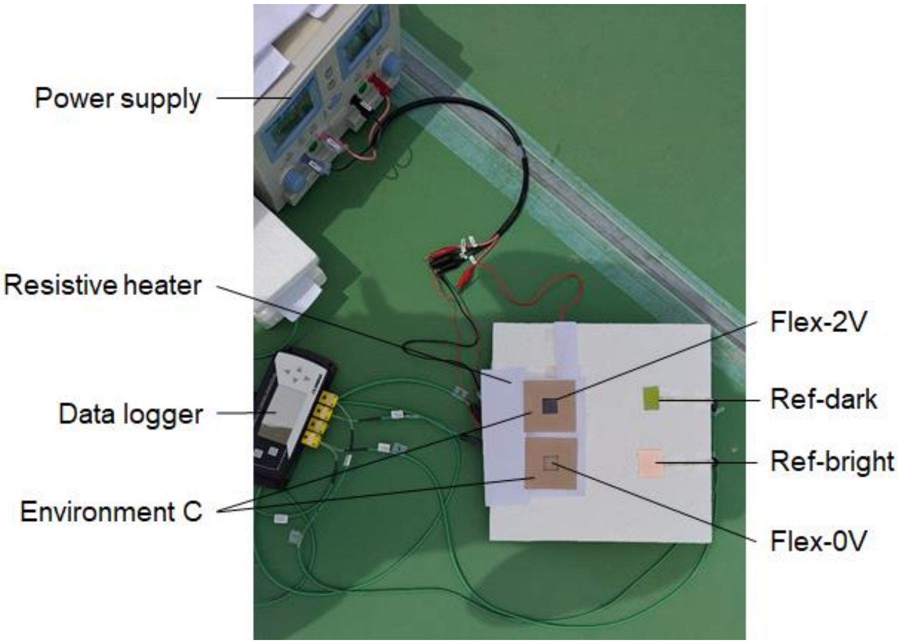

Figure S9. Photographic image for outdoor measurement setup.

Note S7: Additional outdoor measurement and climate conditions

To evaluate the radiative cooling of high-emissive camouflage devices, we conducted additional outdoor measurements as shown in Fig. S10(a). For comparison, we designed two reference samples (Ref-bright and Ref-dark) that absorb sunlight similar to rigid camouflage device but exhibit low emittance in mid-IR (MIR) range (Fig. S10(b)). The structure of reference samples is multi-layer and their stacking materials and thickness are as follows: (1) Ref-0V: Ge(15 nm)/Al(100 nm)/Si wafer(substrate), (2) Ref-2V: ZnS(42 nm)/Ge(9 nm)/ZnS(29 nm)/Cr(20 nm)/ZnS(93 nm)/Al(100 nm)/Si wafer(substrate). Under AM1.5 condition, the absorbed solar irradiance of Ref-bright, Ref-dark, Rigid-0V, and Rigid-2V are 395 Wm^{-2}, 863 Wm^{-2}, 428 Wm^{-2}, and 872 Wm^{-2}, respectively. The climate condition in measurement day is illustrated in Fig. S10(c). Figure S10(d, e) shows the measured temperature, indicating that Rigid-0V (Rigid-2V) is cooler in average of 6.6 K (8.1 K) than Ref-bright (Ref-dark).

In the meantime, Figure S11 illustrates the climate conditions of the outdoor measurements, which identifies the camouflage performance described in the manuscript. Also, Figure S12 displays the actual temperature data measured by thermocouple for rigid camouflage devices and target environments.

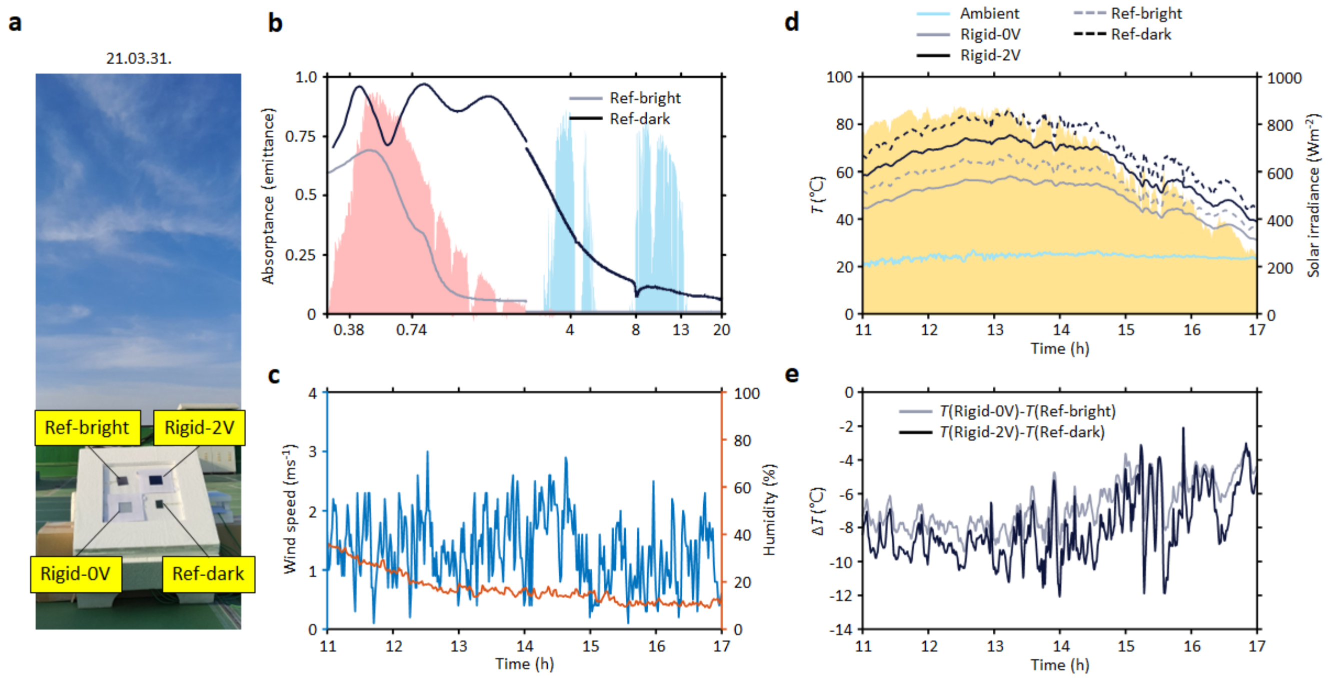

Figure S10. Additional outdoor measurement. (**a**) Photographic image of measurement setup. (**b**) Spectral absorptance (emittance) of reference samples. (**c**) Atmospheric conditions. (**d**) Temperature of rigid camouflage devices and reference samples, and (**e**) their difference depending on voltage.

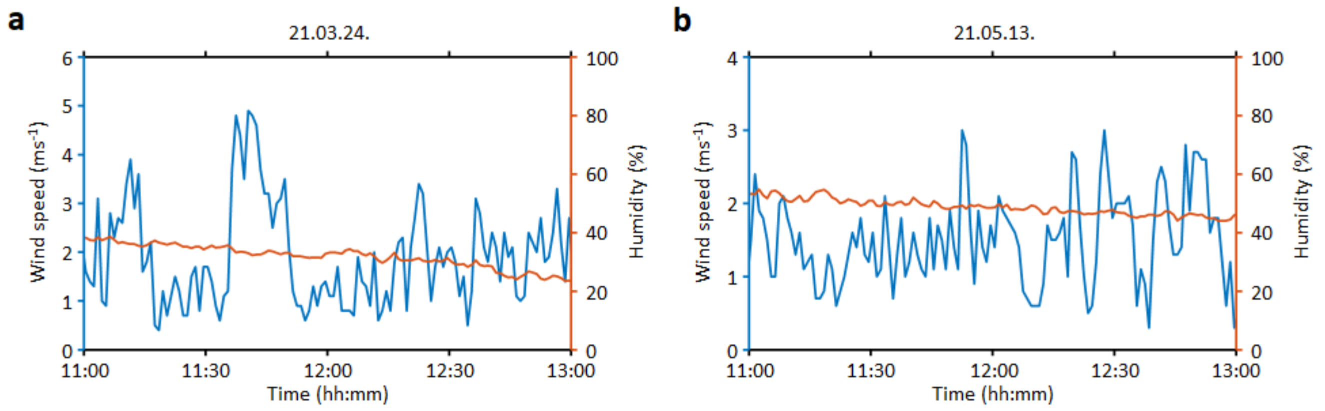

Figure S11. Atmospheric conditions in measurement day for (a) flexible and (b) rigid camouflage devices.

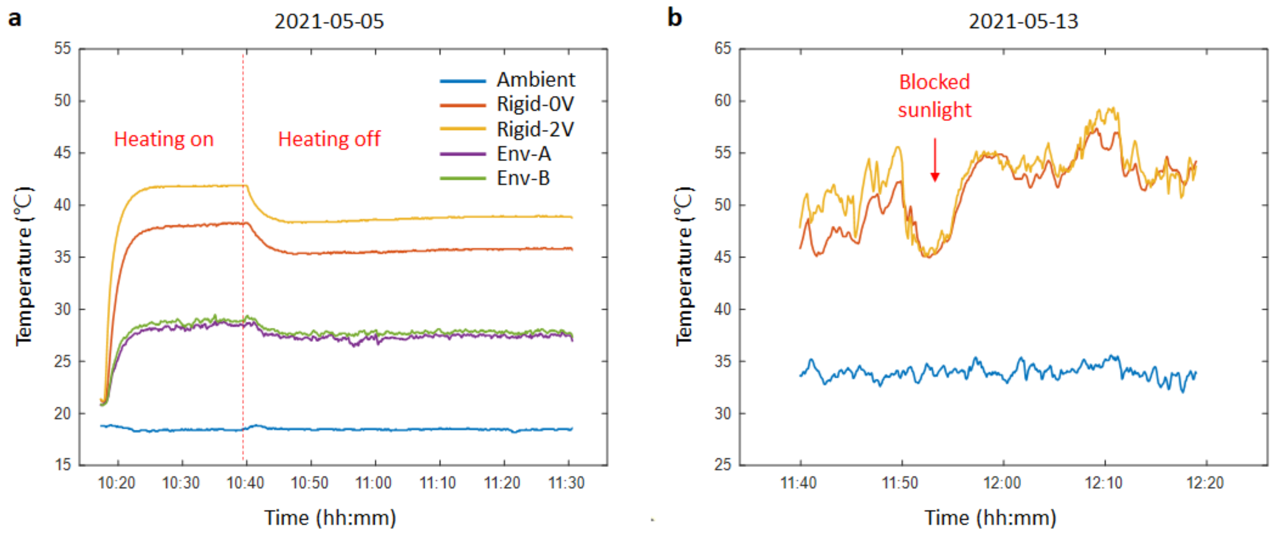

Figure S12. Actual temperature data measured by thermocouples in (a) 2021-05-05 and (b) 2021-05-13.

Note S8: Schematic for fabrication process

The schematic of fabrication process described in Method section is shown in Fig. S13.

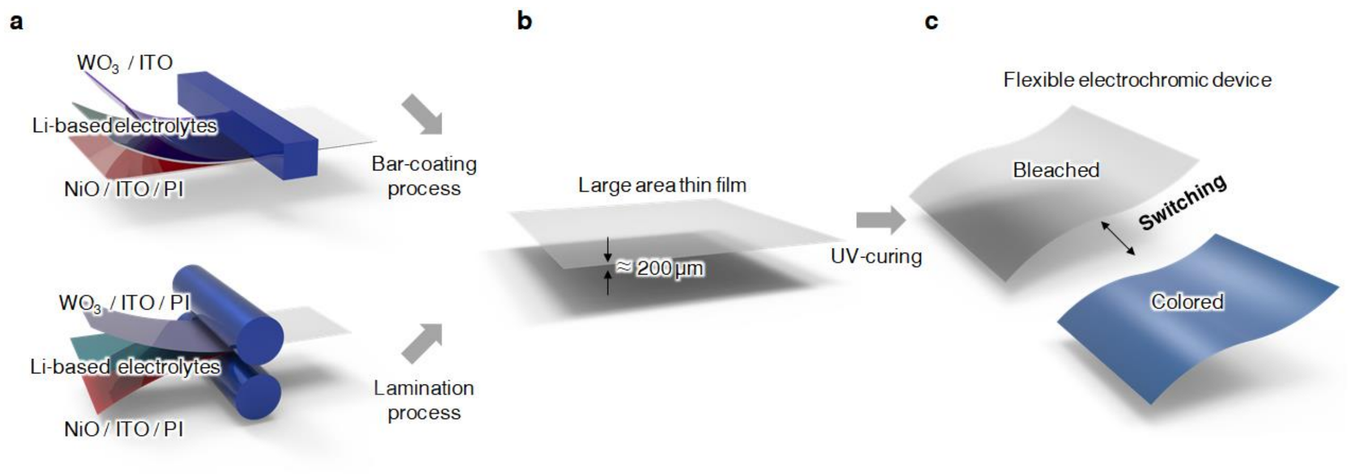

Figure S13. Fabrication process for flexible and rigid camouflage devices.

Supplemental references

[1] Li, W., Shi, Y., Chen, Z., & Fan, S. (2018). Photonic thermal management of coloured objects. *Nature communications*, 9(1), 4240.
[2] Min, S., Jeon, S., Yun, K., & Shin, J. (2022). All-color sub-ambient radiative cooling based on photoluminescence. *ACS Photonics*, 9(4), 1196-1205.
[3] McDowell, M. L., Bruland, G. L., Deenik, J. L., Grunwald, S., & Knox, N. M. (2012). Soil total carbon analysis in Hawaiian soils with visible, near-infrared and mid-infrared diffuse reflectance spectroscopy. *Geoderma*, 189, 312-320.
[4] Cañasveras, J. C., Barrón, V., Del Campillo, M. C., Torrent, J., & Gómez, J. A. (2010). Estimation of aggregate stability indices in Mediterranean soils by diffuse reflectance spectroscopy. *Geoderma*, 158(1-2), 78-84.
[5] Tang, B. H., Wang, J., Li, Z. L., Wu, H., & Tang, R. (2015, December). Measurements of natural surface emissivity with portable Fourier transform infrared spectroradiometer. *In International Conference on Intelligent Earth Observing and Applications* 2015 (Vol. 9808, pp. 381-387). SPIE.
[6] Mattar, C., Santamaría-Artigas, A., Ponzoni, F., Pinto, C. T., Barrientos, C., & Hulley, G. (2019). Atacama Field Campaign: laboratory and in-situ measurements for remote sensing applications. *International Journal of Digital Earth*, 12(1), 43-61.
[7] Sharma, G., Wu, W., & Dalal, E. N. (2005). The CIEDE2000 color-difference formula: Implementation notes, supplementary test data, and mathematical observations. *Col. Res. Appl.* 30(1), 21-30.
